# miRNA Profiling of Developing Rat Retina in the First Three Postnatal Weeks

**DOI:** 10.1007/s10571-023-01347-3

**Published:** 2023-04-21

**Authors:** Péter Urbán, Etelka Pöstyéni, Lilla Czuni, Róbert Herczeg, Csaba Fekete, Róbert Gábriel, Andrea Kovács-Valasek

**Affiliations:** 1 János Szentágothai Research Centre, University of Pécs, Pecs, Hungary; 2 Experimental Zoology and Neurobiology, University of Pécs, Pecs, Hungary; 3 Department of General and Environmental Microbiology, University of Pécs, Pecs, Hungary

**Keywords:** Retina, Postnatal development, miRNA profiling, IonTorrent PGM, qPCR, DIANA

## Abstract

The morphogenesis of the mammalian retina depends on the precise control of gene expression during development. Small non-coding RNAs, including microRNAs play profound roles in various physiological and pathological processes via gene expression regulation. A systematic analysis of the expression profile of small non-coding RNAs in developing Wistar rat retinas (postnatally day 5 (P5), P7, P10, P15 and P21) was executed using IonTorrent PGM next-generation sequencing technique to reveal the crucial players in the early postnatal retinogenesis. Our analysis reveals extensive regulatory potential of microRNAs during retinal development. We found a group of microRNAs that show constant high abundance (miR-19, miR-101; miR-181, miR-183, miR-124 and let-7) during the development process. Others are present only in the early stages (miR-20a, miR-206, miR-133, miR-466, miR-1247, miR-3582), or at later stages (miR-29, miR-96, miR-125, miR-344 or miR-664). Further miRNAs were detected which are differentially expressed in time. Finally, pathway enrichment analysis has revealed 850 predicted target genes that mainly participate in lipid-, amino acid- and glycan metabolisms in the examined time-period (P5–P21). P5–P7 transition revealed the importance of miRNAs in glutamatergic synapse and gap junction pathways. Significantly downregulated miRNAs rno-miR-30c1 and 2, rno-miR-205 and rno-miR-503 were detected to target Prkx (ENSRNOG00000003696), Adcy6 (ENSRNOG00000011587), Gnai3 (ENSRNOG00000019465) and Gja1 (ENSRNOG00000000805) genes. The dataset described here will be a valuable resource for clarifying new regulatory mechanisms for retinal development and will greatly contribute to our understanding of the divergence and function of microRNAs.

**Supplementary Information:**

The online version contains supplementary material available at 10.1007/s10571-023-01347-3.

## Introduction

Vertebrates retina is a multi-layered tissue that is formed as a consequence of a series of sequential cellular events during development: neural progenitor proliferation, cell fate specification, neuronal migration, neurite outgrowth and pathfinding, then eventually the formation of synaptic connections (Amini et al. 2018). Seven principal cell types are generated from a pool of multipotent retinal progenitor cells (RPCs) in a conserved sequence: retinal ganglion cells (RGCs) (embryonic day (E) 9-postnatal day (P) 2), then horizontal cells (E10–E15) and cones (E10–E20) followed by the majority of the amacrine cells (E10–P1) at embryonic state. The remaining amacrine cells and most of the rod photoreceptors (E16–P12) as well bipolar cells (E20–P12) and Müller glial cells (E18–P12) are generated postnatally (Rapaport et al. 2004; Reese 2011; Bassett and Wallace 2012; Hoon et al. 2014). Based on morphologies, physiological properties, and/or sublaminar positions of all major types of retinal neurons can be divided into two or more subtypes, except for rods. Amacrine cells and RGCs are known as the most diversified cell types (Masland 2004; Amini et al. 2018; Sharma et al. 2014).

In order to reach the cell number characteristic of the mature retina, each neuronal cell types have a well-characterized, unique proliferative peak. While postnatally generated cells have a normal or “bell-shaped” curve kinetics of cell production, the early generated cells can be considered as “plateau” shape (Rapaport et al. 2004). During postnatal development, amacrine cells show proliferation peaks on day P1, rod photoreceptors from birth to day P6, while Müller and bipolar cells show proliferation peaks on day P6 followed by a rapid, steady decrease until the opening of the eye, on days P12–P13 (Bagnoli et al. 2003; Bassett and Wallace 2012; Reese and Colello 1992; Rapaport et al. 2004). The development of the morphological characteristics of each cell type in the rat retina lasts from day P10 until day P21 (Johansson et al. 2000).

The migration and differentiation of retinal neurons into the appropriate layer is key for assembling the laminar architecture. Subsequently synaptic connections and functional neuronal circuits are formed. The cells of the outer neuroblast layer are only arranged in the outer and inner nuclear layer separated by the outer plexiform layer (OPL) around P6–P8 (Bagnoli et al. 2003; Amini et al. 2018). In the outer retina, photoreceptors contact horizontal cells and bipolar cells, while retinal ganglion cells synapse their presynaptic partners, the amacrine and bipolar cells in the inner retina forming the inner plexiform layer (IPL). Although synaptogenesis in the IPL begins on P3, while in the OPL on P5, the characteristics of a mature cell only occur when the surviving cells enter the appropriate layer. After migration from P7 on the first synaptic vesicles also appear. The expression of receptors, transporters and synaptic proteins are crucial in synaptic transmission (Bagnoli et al. 2003).

Recently, non-coding RNAs especially microRNAs are deeply researched as crucial factors that are members of the complex gene regulation network involved in generating retinal cell diversity (Xu et al. 2007; Sundermeier and Palczewski 2012; Xiang 2013). Now it is well accepted that retinal disorders at least partially have been connected to abnormal miRNAs expression such as diabetic retinopathy, retinitis pigmentosa, retinoblastoma and age-related macular degeneration (Maiorano and Hindges 2012; Andreeva and Cooper 2014). It is also important to know that studies on Dicer ablated animals demonstrated selective loss of miRNAs which caused developmental changes. In conditional knock-out Dicer retinas the early born cell types such as RPCs were increased and prolonged, while if late RPC markers including Sox9 and Ascl1 were not expressed, late-born cell types including rods and Müller cells were not generated (Gao 2010; Georgi and Reh 2010; Hackler et al. 2010).

Approximately 1% of the human genome consists of miRNA genes (Andreeva and Cooper 2014). The detection of miRNA molecules nowadays is still very challenging because of their low abundance, small size and the high sequence similarities. However, the improvement in technology over the last decade offers new, highly sensitive methodologies to reveal the existing fine-tuning machinery. Several methods are available to qualify or quantify miRNA expression such as in situ hybridisation, Northern blotting, quantitative reverse transcription polymerase chain reaction (qRT-PCR), microarrays, bead-based flow-cytometry and small-RNA sequencing. While most of them support a miRNA-specific detection, the small RNA-sequencing methodology offers the only way to detect all miRNAs present via a high throughput technics (Pritchard et al. 2012; Kolanowska et al. 2018).

Although the structural development of the vertebrate retina has long been researched, knowledge on miRNAs is still very limited. Here, we describe the small RNA profile of postnatal retina in Wistar WU albino rat by Ion-Torrent PGM sequencing technology. These data provide new insight into the postnatal retinogenesis. Moreover, due to the possible similarity, our results may become a useful resource for developmental biology especially in small RNA studies of other sensory organs.

## Methods

### Animals

Animal handling, housing and experimental procedures were reviewed and approved by the ethics committee of University of Pécs (BAI/35/51–58/2016; PTE/43902/2016). To determine the experimental sample sizes a G-power analysis was conducted (Additional file 1). All efforts were made to minimize pain. Animals were housed as families (female + pups)/cage distribution, not separated by gender and without the presence of an adult male. We kept max. two adult rats in a box (T3-H type, 378 × 217 × 180 mm polycarbonate box (Acéllabor Kft., Budapest, Hungary). Animals were maintained on a 12:12 h light/dark cycle and were provided with food and water ad libitum. The day of birth is recorded as postnatal day 0 (P0). The animals were randomly allocated to the groups (P5, P7, P10, P15, P21). Wistar WU rats were anesthetized by inhalation using Forane (Abbott Laboratories, Budapest Hungary) prior to sacrifice at the same hour of the day to avoid circadian variation. We had five groups (P5, P7, P10, P15, P21) and carried out two methodologies (sequencing and qPCR, see details later) to examine the retinal development changes of these animals. For sequencing experiment we used the following number of animals: P5 *n* = 3; P7 *n* = 6; P10 *n* = 3; P15 *n* = 3; P21 = 9. By the end of sequencing, biological sample library originated from RNAs of three individuals. Biological replicates mean at least two different biological samples, all of them originated from RNAs of three individuals. In that case the sequencing outcomes represents finally the miRNA profiles of at least six animals, while technical replicate came from the same barcoded library that was sequenced on another chip. In that case the RNA profile originated from the same three animals: their RNAs were pooled for library preparation and this library was barcoded, then used for template preparation, and finally this template was sequenced on the appropriate chip of the ION Torrent PGM Machine. Similar pooling method was applied by Hackler et al. For qPCR validation the number of the animals was 6, that contained in all cases the samples that were used for sequencing methodology. Each animal was measured in three replicates.

The purpose of this exploratory study was mainly to find over/under expressed small non-coding RNAs in consecutive time points in the developing retinas. As we have relatively few time points (3–5), we handled this simple time course experiment similar to an experiment with several groups (see limma, 9.6.1 guide in R statistic environment).

### miRNA Isolation

Total RNA was extracted from the retinal tissues of various developmental stages using NucleoSpin miRNA kit (Macherey–Nagel, Düren, Germany, Cat: 740971.50) following manufacturer’s instructions. The miRNA concentration was assessed using Qubit microRNA Assay Kit (ThermoFisher Scientific, MA, USA, Cat: Q32880). The RNA integrity number (RIN), an algorithm for judging the integrity of RNA samples, were evaluated using Agilent 2100 Bioanalyzer (Agilent Technologies, Santa Clara, California, USA, Cat: G2939BA) following the manufacturing instruction of the RNA 6000 Nano kit (Agilent Technologies, Santa Clara, California, USA, Cat: 5067-1511) and RIN > 7 was considered acceptable. MicroRNAs was also determined using the Agilent Small RNA Kit (Agilent Technologies, Santa Clara, California, USA, Cat: 5067-1548) to have deeper view in the 10–40-nucleotide size range.

### Small RNA Sequencing

Small RNA library construction from pooled retinal samples (*N* = 3, in each age group) were carried out according to the Ion Total RNA-Seq Kit v2 (ThermoFisher Scientific, MA, USA, Cat: 4475936) protocol (Revision E) with slight modification and the experimenters were blinded during the experimental procedures. Enzymatic reaction was performed in half reaction volume, while cleaning procedures were executed with the accurate final volume according to the protocol.

3 μg RNA per three biological replicates in each age group was pooled and enriched for small RNA applying the Magnetic Bead Cleanup Module. Eluted small RNA from the beads was concentrated and used for the hybridisation step at 65 °C for 10 min and 16 °C for 5 min. Ligation reagents (6 μL) were then added and the samples were incubated at 16 °C for 24 h.

After ligation, reverse transcription (RT) was performed on a Veriti™ 96-Well Thermal Cycler (ThermoFisher Scientific, MA, USA, Cat: 4452300): 8 μL RT master mix was added to the ligated RNA samples (10 μL), tubes were incubated at 70 °C for 10 min, and then snap-cooled on ice, the RT enzyme (2 μL of 10× SuperScript® III Enzyme) was added, and reactions (20 μL) were incubated at 42 °C for 30 min. cDNA from the RT reaction was purified and size-selected according to the protocol of Magnetic Bead Cleanup Module and eluted in 12 μL of nuclease-free water.

For amplifying the cDNA, 3 μL of the purified cDNA was combined with 0.5 μL of Ion Xpress™ RNA 3′ Barcode Primer, 0.5 μL of Ion Xpress™ RNA-Seq Barcode BC primer (choose from BC01 to BC16 for different samples, ThermoFisher Scientific, MA, USA, Cat: 4475485), 22.5 μL of Platinum PCR SuperMix High Fidelity, reaction mix (26.5 μL) was then amplified using the following protocol: 94 °C for 2 min; (94 °C for 30 s, 50 °C for 30 s, and 68 °C for 30 s) for 2 cycles; (94 °C for 30 s, 62 °C for 30 s, and 68 °C for 30 s) for 16 cycles; 68 °C for 5 min. The amplified DNA for each sample was completed to 53 μL with nuclease-free water and samples were purified using the Magnetic Bead Cleanup Module and eluted in 15 μL of nuclease-free water.

The quality and quantity of the library was detected following the manufacturer’s recommendations of High Sensitivity DNA Chip of Agilent Bioanalyzer 2100 (Agilent Technologies, Santa Clara, California, USA, Cat: 5067-4626). The ratio of miRNA ligation products in total ligation products was calculated using the formula for Barcoded libraries: [Area (94–114 bp)] ÷ [Area (50–300 bp)]. The molar concentration of the barcoded library was determined using size range 50–300 bp, then diluted to a final concentration of ~ 20 pM following the manufacturer’s protocol. Equal volumes of three or six diluted barcoded libraries were combined for the next steps depending on the chip type.

For template preparation, the diluted library (15 μL) was used to generate template positive Ion Sphere™ Particles (ISPs) containing clonally amplified DNA. EmulsionPCR (emPCR) was carried out on the Ion OneTouch™ 2 System (ThermoFisher Scientific, MA, USA, Cat: 4474779) with the Ion PGM™ Hi-Q View OT2 200 Kit (ThermoFisher Scientific, MA, USA, Cat: A29900) following the recommended protocol. Template-positive ISPs were enriched with the Ion OneTouch™ ES (ThermoFisher Scientific, MA, USA, Cat: 4469495) following the manufacturer’s recommendations.

The Ion 316 or 318 ™ Chip v2 (ThermoFisher Scientific, MA, USA, Cat: 4488149, 4488146) was used for sequencing on the Ion Torrent PGM™ instrument (ThermoFisher Scientific, MA, USA, Cat: 4462921) according to the protocol of Ion PGM™ Hi-Q View Sequencing Kit (ThermoFisher Scientific, MA, USA, Cat: A30044). Sequencing primer and Control Ion Spheres™ Particles of the Ion PGM sequencing kit were added to the enriched, template-positive ISPs. After annealing the sequencing primer, sequencing polymerase was added and a final volume of 30 μL was loaded onto the chips.

To assess reliability of miRNA workflow solution in 316 v2 chip (as Yao et al. 2012), two independent P7 samples were assayed (biological replicates) and P21 sample were sequenced as technical replicates in every sequencing procedure. Some samples were also sequenced in 318 v2 chip (P5, P10, P15 and P21) as biological replicate.

### Computational Analysis

Ion Torrent Suite Platform was used to trim the raw sequence data and remove any residual sequencing adapter fragments that remained on the 5′ or 3′ ends. Reads were mapped to the non-coding RNAs from ENSEMBL [Rnor_6.0 (GCA_000001895.4)] using manufacturers’ alignment tools for PGM, TMAP algorithm. These aligned BAM (Binary Alignment Map) files were further processed in Galaxy Web-based platform (Afgan et al. 2018) via Cufflinks, Cuffmerge and Cuffdiff (Version 2.2.1.3) application (Trapnell et al. 2010). The expression level of individual transcripts was calculated fragments per kilobase million (FPKM) method in Cufflinks, while geometric library method with 25 of average fragment length was applied in Cuffdiff. Pearson’s correlation was used to assess the correlation between read counts per miRNA of biological replicates. Further ascertainment of the most important miRNAs were based on statistical analysis in R Studio Software environment (RStudio Team 2015).

DIANA miRPath 3.0 was used for miRNA target and pathway analysis (Vlachos et al. 2015). Experimentally validated miRNA targets were identified using DIANA-TargetScan and the Genes Union option (an a posteriori method) was used for pathway analysis. The integrated Fisher’s Exact test followed by FDR adjustment were used for statistical analysis. Enriched pathways from the KEGG database were exported from the tool along with the corresponding FDR-corrected *P* values. A significance threshold of FDR = 0.05 was applied to the corrected *P* values.

### Validation of the Sequencing Results

TaqMan® MicroRNA Assays (ThermoFisher Scientific, MA, USA, Cat: 4427975) were applied to validate the miRNA expression profiles. Isolated RNA from six retina in each age group were reverse-transcribed with TaqMan MicroRNA Reverse Transcription Kit (ThermoFisher Scientific, MA, USA, Cat: 4366596) according to the manufacturer’s instructions. Briefly, 15 μL of final volume contained 5 μg purified total RNA (10 ng), 10× RT buffer, 100 mM dNTPs, 50 U/μL MultiScribe Reverse Transcriptase (MuLV), 20 U RNase inhibitor and 3 μL 5× stem-loop RT-primers. The reactions were incubated in Veriti thermal cycler (ThermoFisher Scientific, MA, USA, Cat: 4452300) at 16 °C for 30 min, 42 °C for 30 min, 85 °C for 5 min and then held in 4 °C. RT-PCR was then preformed using the TaqMan Universal PCR Master Mix and the specific primers from TaqMan MicroRNA Assay specific for each miRNA. U6 small nuclear RNA (U6 snRNA) (ThermoFisher Scientific, MA, USA, assay ID 001973) was used as an endogenous control. Studied miRNA were miRNA-9 (ThermoFisher Scientific, MA, USA, assay ID 000583) and miRNA-23 (ThermoFisher Scientific, MA, USA, assay ID000399). In a 20-μL PCR reaction, 1.33 µL of cDNA was added to 10 μL of TaqMan Universal PCR master mix II (ThermoFisher Scientific, MA, USA, Cat: 4440043) and 1.0 μL of the 20 × TaqMan miR-specific primers and probe mix. The reactions were incubated in a 96-well optical plate at 95 °C for 10 min followed by 40 cycles of 95 °C for 15 s and 60 °C for 60 s, using a Step One Plus Detection system (ThermoFisher Scientific, MA, USA, Cat: 4376600). Negative controls (without template) were also established and all experiments were run in triplicate. Relative quantification was performed by the 2^−ΔΔCT^ method (Livak and Schmittgen 2001) and P21 was used as reference.

### Availability of Data

The gene expression profiling data discussed in this publication have been deposited in the NCBI Gene Expression Omnibus and are accessible through GEO Series accession number GSE159168.

## Results

**Fig. 1:**
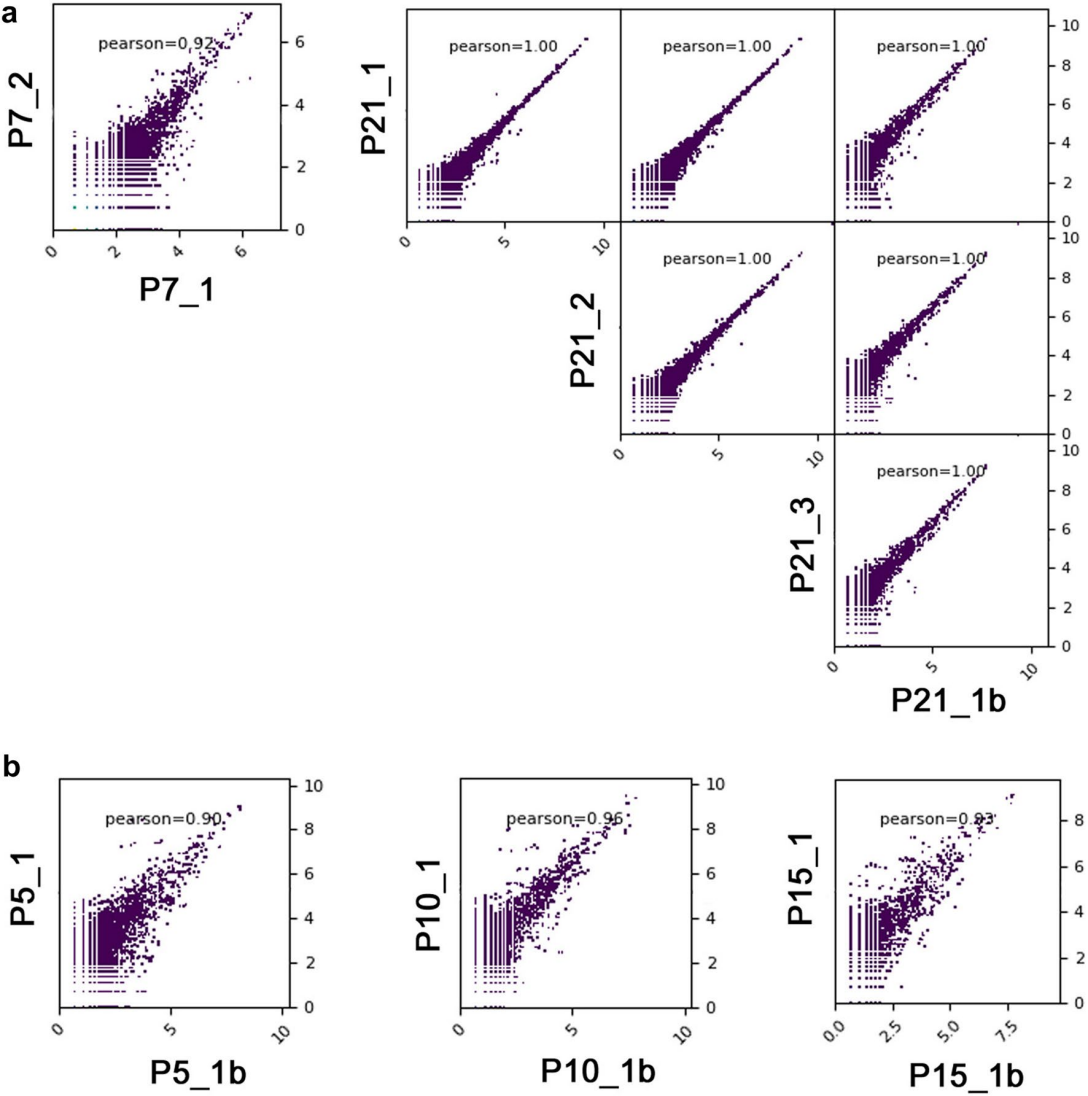
Pearson correlation coefficients for global miRNA expression profiles among all samples. **A** Technical replicates, **B** Biological replicates

To reveal the main miRNAs quantitative alterations during the early postnatal retinal development total RNA samples were extracted from Wistar WU rat retinal tissues of five developmental stages (P5, P7, P10, P15, P21). In order to monitor the general quality of extracted RNA samples RIN was calculated by Agilent Bioanalyzer Software. High quality and low degradation samples (RIN ≥ 7) were only processed further. To evaluate the reproducibility of the experimental procedures in 316 v2 chip two independent P7 samples were assayed (biological replicates) and P21 samples were sequenced as technical replicates in every sequencing procedure. The miRNA levels between the two independent P7 tissue samples and every P21 samples were characterized by a high Pearson’s correlation coefficient (Pearson *r* = 0.92 and 1.00, respectively), that means high reproducibility of the sequencing (Fig. 1A). Some samples were also sequenced in 318 v2 chip (P5, P10, P15 and P21; as biological replicate) also with a high Pearson’s correlation coefficient (Fig. 1B). Results were averaged to reduce experimental errors. In 316 v2 chip we obtained in total 143,796,965 bases and in average 12,182,661 reads for each sample after removal of low-quality reads and contaminants, with the peak length of each sample at about 24 nt (Additional file 2).

RNA reads were annotated based on their sequences mapped onto *Rattus norvegicus* reference from ENSEMBL (Rnor_6.0 (GCA_000001895.4)) (in average 420 178 reads/sample), and their relative abundances were determined by their counts, normalized to the number of RPKM methods. To minimize the false positive signal, only reads that were detected in both 316 and 318 v2 sequencings (biological and technical replicates) were used for further bioinformatics analysis. Principal component analysis (PCA) revealed partial separation of samples based on postnatal day of development as shown in Additional file 3.

Based on the non-coding RNA classification methods of ENSEMBL our data generated by sequencings were classified into five functionally important categories including ribosomal RNAs (rRNAs), as well as small RNAs such as microRNAs, snoRNAs and snRNAs (Table 1).

**Table 1:** Classification of expressed non-coding RNAs detected by IonTorrent PGM sequencing as time-scale manner

Comparison	rRNA	miRNA	snRNA	snoRNA
P5_P7	97	643	623	686
P7_P10	97	630	603	668
P10_P15	91	610	580	621
P15_P21	96	627	617	680

It is well known the rRNAs playing important roles in the protein synthesis machinery, the highest rRNA number was 97 and the lowest value was 91 at the comparison of P10 and P15. The total expression levels for small RNAs that contribute to the biogenesis of rRNAs or to the protein synthesis, mainly characterized by the same tendency during the development period. Slight decrease of the number of non-coding RNAs at P10 suggests an important role of regulation of protein synthesis for the retinal synaptogenesis.

**Fig. 2:**
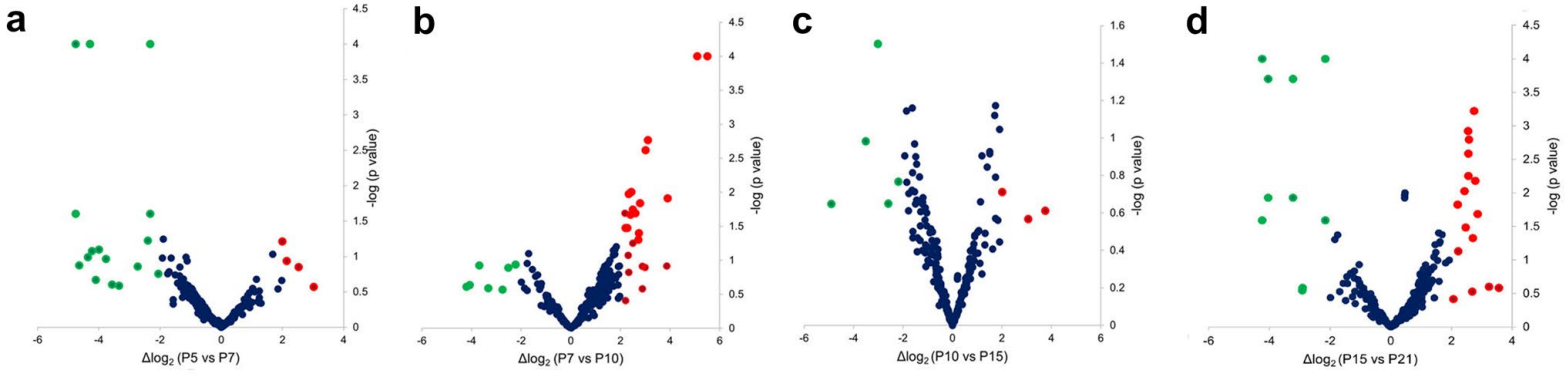
Volcano plots of differentially expressed miRNAs comparing consecutive time points in Wistar rat retina. **A** Postnatally day of 5 (P5) vs postnatally day of 7 (P7), **B** P7 vs P10, **C** P10 vs P15, **D** P15 vs P21. The red circles represent upregulated miRNAs and the green circles indicate downregulated miRNAs (twofold-change). *X*-axis indicate difference in expression level on a log2 scale, whereas the *y*-axis represents corresponding *P* values (Student’s *t*-test) on a negative log scale; more lateral and higher points mean more extensive and statistically significant differences, respectively. The fully colored points (red or green) indicate *P*-value of less or equal than 0.05

Focusing on the known miRNAs in non-coding RNAs from ENSEMBL, we identified approximately 272 miRNAs in at least one of the five developmental stages. The expression profile of miRNAs could provide indication of their potential functions during development. We observed that although there was no obvious difference in the total number of miRNAs detected in retina during development process, the expression level of different miRNAs in retina was very dynamic over stages (Fig. 2). The most abundant miRNAs at each developmental stages are collected in Table 2.

**Table 2:** The top 20 most abundant miRNAs at each developmental stages

P5	P7	P10	P15	P21
rno-miR-376c	rno-miR-376c	rno-miR-106a	rno-miR-106a	rno-miR-376c
rno-miR-106a	rno-miR-106a	rno-miR-376c	rno-miR-376c	rno-miR-106a
rno-miR-206	rno-miR-206	rno-miR-206	rno-miR-206	rno-miR-206
rno-miR-19a	rno-miR-377	rno-miR-377	rno-miR-124-1	rno-miR-124-1
rno-miR-141	rno-miR-124-1	rno-miR-124-1	rno-miR-129-1	rno-miR-129-1
rno-miR-143	rno-miR-19a	rno-miR-129-1	rno-miR-377	rno-miR-377
let7c-2	rno-miR-143	rno-miR-19a	rno-miR-19a	rno-miR-320a
rno-miR-377	rno-miR-129-1	let7c-2	rno-miR-34b	rno-miR-19a
rno-miR-124-1	rno-miR-320a	rno-miR-141	let7c-2	rno-miR-3571
rno-miR-152	rno-miR-152	rno-miR-103a1	rno-miR-298	rno-miR-100
rno-miR-129-1	let7c-2	rno-miR-34b	rno-miR-18a	let7c-2
rno-miR-320a	rno-miR-141	rno-miR-18a	rno-miR-141	rno-miR-3570
rno-miR-100	rno-miR-344b-2	rno-miR-298	rno-miR-103a1	rno-miR-18a
rno-miR-103a1	rno-miR-103a1	rno-miR-152	rno-miR-144	rno-miR-144
rno-miR-3570	rno-miR-100	rno-miR-34a	rno-miR-320a	let7b
rno-miR-18a	rno-miR-3570	rno-miR-29b1	rno-miR-222	rno-miR-29b1
rno-miR-34b	rno-miR-18a	rno-miR-143	rno-miR-34a	rno-miR-103a1
rno-miR-298	rno-miR-222	rno-miR-320a	rno-miR-29b1	rno-miR-222
rno-miR-29b1	rno-miR-29b1	rno-miR-222	rno-miR-152	rno-miR-19b1

The differentially expressed miRNAs were collected on consecutive time points with *P* < 0.05 (Additional file 4). Interestingly, only downregulated miRNAs reached the cut-off at P5 vs. P7 and P10 vs. P15, while mainly upregulated miRNAs were detected at P7 vs. P10. There are some miRNAs that show constant high abundance during the development process such as miR-19, miR-101; miR-181, miR-183, miR-124 and let-7. While there are miRNAs that appear as most abundant only in the early stages such as miR-20a, miR-206, miR-133b, miR-466, miR-1247 or miR-3582, others are characteristic with high abundance at later stages or increasing with development for example miR-29b, miR-96, mir-125, miR-344 or miR-664.

**Fig. 3:**
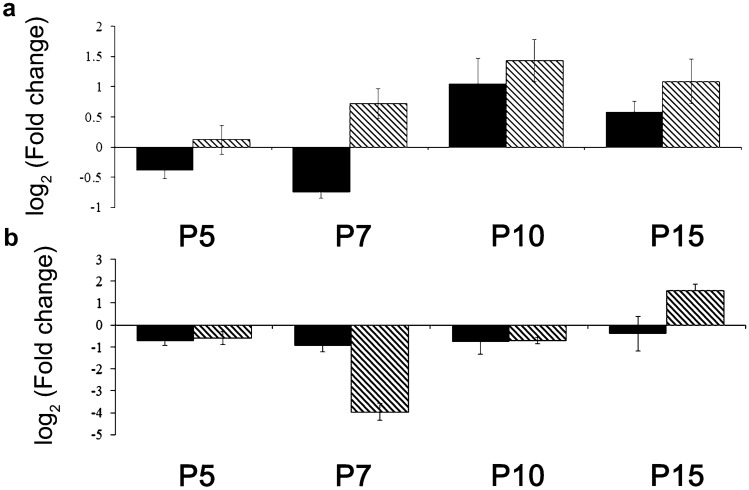
Comparison results of rno-mir-RNA sequencing (black) and qPCR (striped). **A** Fold changes in rno-mir-9 and **B** rno-mir-23 levels (mean ± SEM) identified during retinal postnatal development (Pöstyéni et al. 2021a, b)

To further validate the sequencing results by quantitative polymerase chain reaction (qPCR) we choose miR-9 and miR-23 which based on our previous investigations and literature search seemed to have major importance during postnatal retinal development (Leucht et al. 2008; Arora et al. 2010; Gao 2010; La Torre et al. 2013; Qi 2016; Pöstyéni et al. 2021a, b).The relative expression level of miR-9 exhibited the upregulated pattern while that of mir-23 exhibited the downregulated pattern compared to P21. In addition, the expression patterns of miRNAs revealed by qPCR were mostly consistent with sequencing results (Fig. 3) with some exceptions. For example, miRNA-9 at P5 and P7 or mir-23 at P15, when the sequencing results suggest downregulation compared to P21, while qPCR data propose minor upregulations. Despite of these minor discrepancies between qPCR and deep-sequencing results, our data are highly correlated and accurate in detection and quantification of the relative expression levels of miRNAs.

### Prediction and Functional Analysis of Differentially Expressed miRNA Target Genes

**Fig. 4:**
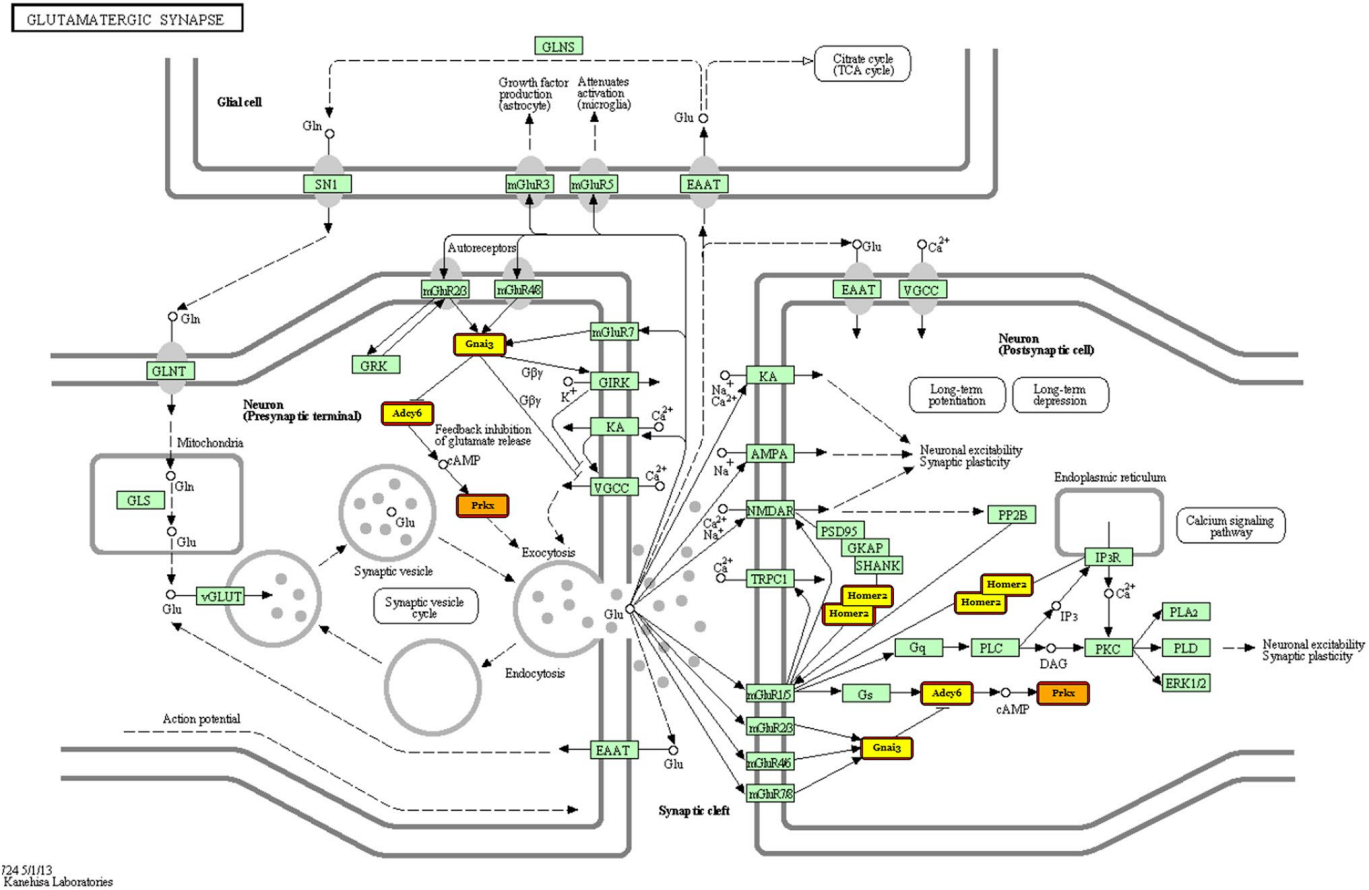
Flow chart of glutamatergic synapse pathway. Highlights are presented genes that targeted by miRNAs identified by IonTorrent PGM sequencing. The genes targeted by more than one miRNA are colored by orange, while the genes targeted by a single miRNA are colored in yellow. Prkx (ENSRNOG00000003696), Adcy6 (ENSRNOG00000011587), Gnai3 (ENSRNOG00000019465) and Homer2 (ENSRNOG00000019297)

**Fig. 5:**
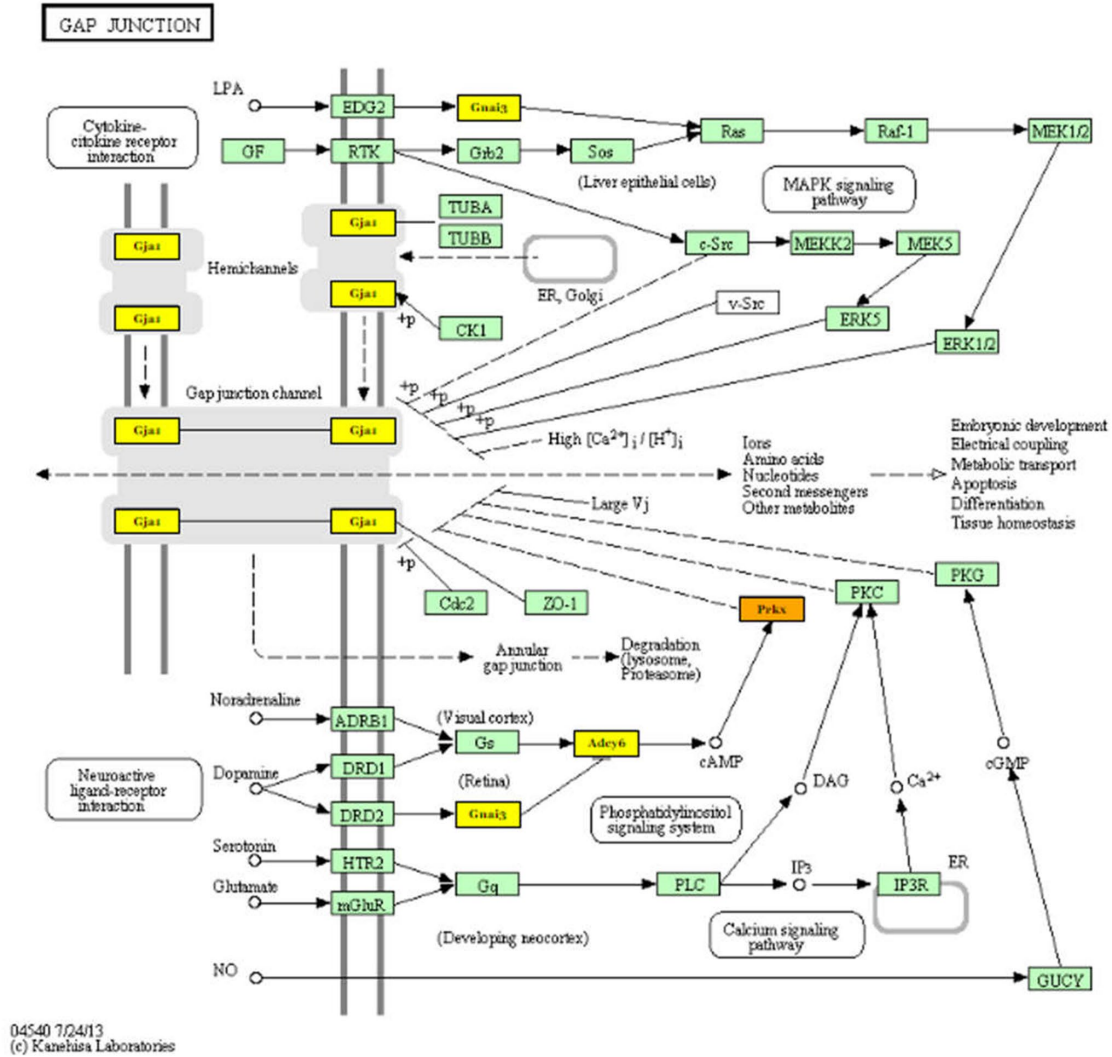
Flow chart of gap junction synapse pathway. Highlights are presented genes that targeted by miRNAs identified by IonTorrent PGM sequencing. The genes targeted by more than one miRNA are colored by orange, while the genes targeted by a single miRNA are colored in yellow. Prkx (ENSRNOG00000003696), Adcy6 (ENSRNOG00000011587), Gnai3 (ENSRNOG00000019465) and Gja1 (ENSRNOG00000000805)

To gain insight into the roles of the differentially expressed miRNAs during postnatal-development of the retina, DIANA web-tool was used to predict potential target genes and to apply pathway enrichment analysis. This enrichment analysis identifies pathways significantly enriched with genes belonging to all genes targeted by at least one selected miRNA. For differentially expressed miRNAs, 850 predicted target genes were annotated with function. It is important to know that each miRNA can have different targets, and different miRNAs can have the same target gene. Between enriched pathways targeted by miRNAs cut off for the value of log2 fold change was more or less than ± 1.5 (*P* < 0.05) there were as follows: lipid metabolisms (such as unsaturated fatty acid, arachidonic acid or glycerophospholipid), amino acid metabolisms (such as valine, leucine, isoleucine or lysin degradation, tyrosine metabolism) and glycan metabolisms in large numbers. The dataset of P5–P7 transition has shown the crucial role in glutamatergic synapse formation (Additional file 5). Among significantly downregulated miRNAs rno-miR-30c1 and 2, rno-miR-205 and rno-miR-503 were detected to target Prkx (ENSRNOG00000003696), Adcy6 (ENSRNOG00000011587), Gnai3 (ENSRNOG00000019465) and Homer2 (ENSRNOG00000019297) genes as shown in Fig. 4 highlighted. Furthermore, the data analysis of P5–P7 transition has also revealed the importance in gap junction (rn04540) KEGG pathways (Additional file 5). In addition to Prkx (ENSRNOG00000003696), Adcy6 (ENSRNOG00000011587) and Gnai3 (ENSRNOG00000019465) genes, Gja1 (ENSRNOG00000000805) appears strongly influence in this pathway (Fig. 5). These results suggest roles of miRNAs in regulating cell metabolism, synapse formation (both chemical and electric) as well as proliferation/survival/migration processes involved in the retinal development process.

## Discussion

Nowadays increasing number of data are generated by sequencing, especially to understand gene expression regulation at miRNA level in retina. However, there is a limited number of studies that focuses on normal developmental events only with the aim to reveal the fine-tuning roles of miRNAs in the gene regulatory network (Xiang 2013; Xu et al. 2007; Georgi and Reh 2010; Arora et al. 2010; Torre et al. 2013; Coolen et al. 2013; Kapsimali et al. 2007). It is already well-known that miRNAs are crucial for physiological and developmental processes in the central nervous system including in the retina (Sundermeier and Palczewski 2012; Maiorano and Hindges 2012; Gao 2010; Hackler et al. 2010; Linsen et al. 2010). Here, we analyzed expression of miRNAs in Wistar albino rat retina tissue during postnatal development and we found that miRNAs showed a wide diversity of expression pattern. Some of them seemed to be preferentially enriched in the 1st days such as rno-mir-20a, rno-mir-133, rno-mir-206, rno-mir-466c, while others exhibited a higher abundance at later days, indicating distinct roles played in controlling the development of late-born retinal cells (rod photoreceptors, bipolar cells and Muller glia) and in maintaining the retinal neurons and/or Muller glia (for example rno-mir-29, rno-mir-96, rno-mir-125, rno-mir-344 (Xu et al. 2007)). Again other miRNAs have shown constant high abundance such as let-7 as the most highly expressed rno-miRNA related to retinal-targets (CLASP2, DMD, DUSP1, ELOVL4, NLK, FZD4, RB1, RDH10, RGS16, SLC25A18; Arora et al. 2007).

There is only a limited number of studies that represented an overall description of spatial and temporal rno-miRNA expression in rodent eye. They revealed cell type-specific enrichments of some rno-miRNAs in the retina, such as rno-mir-29c, rno-mir-30d, rno-mir-96, rno-mir-99b, rno-mir-124a; rno-mir-182; rno-mir-183; rno-mir-184; rno-mir-381 and rno-mir-425 of rod- and cone photoreceptor in outer nuclear layer in adult mice. Others (rno-mir-409-5p, rno-mir-433, rno-mir-541 and rno-mir-742) have shown amacrine specific staining (Karali et al. 2010). This rno-mir-Neye atlas determined the expression profiles of rno-miRNAs in the lens, cornea and retinal pigment epithelium of the adult mouse eye. However, when we made retinal total cell extract, we were able to detect most of them except rno-mir-29c; rno-mir-182; rno-mir-409-5p and rno-mir-742. The rno-mir-184 was detectable in a meaningful quantity only at P5.

Furthermore, it is also well-established that let-7, rno-mir-125 and rno-mir-9 play an essential role in the developing mouse retina to regulate the transition from early to late stage retinal progenitors (Andreeva and Cooper 2014).

Other studies emphasize the importance of the rno-mir-183/96/182 cluster as a sensory organ specific rno-miRNA. All three of these miRNAs have a similar developmental expression pattern and transcribed as a single polycistronic transcript. The sensory organ specificity of these miRNAs is also supported by sequence analysis of the upstream CpG island containing multiple predicted transcription factor binding sites characteristic of genes expressed in neurosensory cells, such as OTX1, Pou3F2 or Pax5 (Xu et al. 2007; Coolen et al. 2013). In a good agreement with these studies we have found a dramatic increase of rno-mir-183/96/182 by the 10th postnatal day. We have already mentioned that we were able to detect rno-mir-183 and rno-mir-96, but interestingly not rno-mir-182.

Our findings are also broadly in line with the microarray based study of microRNA expression in C57BL/6 mouse eye in the case of rno-miRNAs with increasing or decreasing expression tendency during development in wild-type animals [e.g. declining expression of rno-mir-20; increased of rno-mir-29 or rno-mir-125 (Hackler et al. 2010)]. However, there are some differences comparing the results to the study of Hackler et al. (2010). While in our study rno-mir-16 showed constant high expression with gentle alterations (namely there is a slight decrease from P5 to P7, then a peak at P10 after that again a slight decrease to P21), in the developing C57BL/6 mouse retina this rno-miRNA was revealed with a decreasing tendency. At the same time, in the case of rno-mir-30 an increasing expression pattern was detected in wild-type mouse, our detailed time-scaling of postnatal days was able to detect a fluctuating profile: decreasing tendency to a minimum at P7, after that a sharp increment to P15 and finally a decline at P21 again. Finally, rno-mir-103 was assessed in our study to have a gradually increasing profile, while it was not even mentioned in the developing C57BL/6 mouse retina.

In order to better understand the role of miRNAs miRNA-target interactions have been revealed. DIANA web-tool found their roles in various biological processes. All of our investigations the dataset of P5–P7 transition seems the most exciting from the aspect of retinogenesis. Although the development of the neural retina and visual system, especially synaptogenesis, is complex, studies have shown that the major events correspond to occur at a special period (Rapaport et al. 2004; Reese 2011; Bassett and Wallace 2012; Hoon et al. 2014; Kapsimali et al. 2007).

Our P5–P7 transition dataset seems to underline the possible roles of miRNAs in glutamatergic synapse and gap junction formation. There are remarkable changes at this time in the retina as we mentioned earlier. Formation of the OPL through the rearrangement of the neuroblast layers cells (Bagnoli et al. 2003; Amini et al. 2018), and the proliferation peaks of Müller and bipolar cells have also been observed at that time (Bagnoli et al. 2003; Bassett and Wallace 2012; Reese and Colello 1992; Rapaport et al. 2004) Furthermore the synaptogenesis mainly occurs in the OPL at this time and the first synaptic vesicles appear (Bagnoli et al. 2003). In the neuronal circuitry of the retina chemical transmission is dominated by glutamate and γ-aminobutyric acid (GABA), being the major excitatory and inhibitory neurotransmitters, respectively (Yang 2004). Glutamate is the neurotransmitter of the neurons (photoreceptors, bipolar cells, ganglion cells) of the vertical pathways through the entire retina (Massey 1990; Crooks and Kolb 1992; Thoreson and Witkovsky 1999; Haverkamp and Wässle 2004). Specific vesicular glutamate transporters (vGLUTs) load glutamate into synaptic vesicles. Studies from rodent retina have also revealed that remarkable maturational processes of bipolar cell happen at P6–P8: namely OFF and ON bipolar terminals first invade IPL; OFF-bipolar cell terminals express vGluT1 (P6), before ON-bipolar cell terminals (around P10); and rod bipolar cells also appear early (P6) (Sherry et al. 2003). These events are important because the inception of the ON and OFF pathways segregate at bipolar cell level (Yang 2004).

Although there are no data in the literature about the most of the potential target genes (Prkx, Adcy6, Gnai3, Homer2) predicted by our studies, we assume their essential role in retinogenesis based on the limited number of investigation demonstrate their importance in developmental processes in various tissues especially in the olfactory system (Li et al. 2005; Azaiez et al. 2015; Huang et al. 2016; Guo et al. 2017; Beer-Hammer et al. 2018). Finally, Gja1 [also known as connexin43 (Cx43)] is the most abundant gap junction protein in the central nervous system and is expressed primarily on astrocytes and Müller cells, thus responsible for maintaining the local homeostasis (Kerr et al. 2010; Völgyi et al. 2013).

## Conclusions

The dataset described here will be a valuable resource for getting deeper insight into the gene regulatory network during retina development. Numerous genes are involved in the formation and differentiation of the retina are targeted by various miRNAs thus creating a complex regulatory network to usher the development of the visual system in stage specific manner. So, these data provide useful framework for studying the expression across development or the impact of pharmacological manipulations at a particular developmental stage, especially in P5–P7 transition.

## Supplementary Information

Below is the link to the electronic supplementary material.

Supplementary file1 (PDF 507 kb)

## Data Availability

The gene expression profiling data discussed in this publication have been deposited in the NCBI Gene Expression Omnibus and are accessible through GEO Series Accession Number GSE159168 (https://www.ncbi.nlm.nih.gov/geo/query/acc.cgi?acc=GSM4820829).

## Abbreviations

Cx43: Connexin43
E: Embryonic day
emPCR: EmulsionPCR
IPL: Inner plexiform layer
ISPs: Ion Sphere™ Particles
MuLV: MultiScribe Reverse Transcriptase
OPL: Outer plexiform layer
P0: Postnatal day 0
PCA: Principal component analysis
qRT-PCR: Quantitative reverse transcription polymerase chain reaction
P: Postnatal day
RGCs: Retinal ganglion cells
RPCs: Retinal progenitor cells
RT: Reverse transcription
rRNAs: Ribosomal RNAs
RIN: RNA integrity number
RPKM: The number of reads per kilobase of the exon model per million mapped reads

## References

[CR1] Afgan E, Baker D, Batut B et al (2018) The Galaxy platform for accessible, reproducible and collaborative biomedical analyses: 2018 update. Nucleic Acids Res 46:W537–W544 29790989 10.1093/nar/gky379 PMC6030816

[CR2] Amini R, Rocha-Martins M, Norden C (2018) Neuronal migration and lamination in the vertebrate retina. Front Neurosci 11:742 29375289 10.3389/fnins.2017.00742 PMC5767219

[CR3] Andreeva K, Cooper NGF (2014) MicroRNAs in the neural retina. Int J Genomics 2014:165897 24745005 10.1155/2014/165897 PMC3972879

[CR4] Arora A, McKay GJ, Simpson DAC (2007) Prediction and verification of miRNA expression in human and rat retinas. Investig Ophthalmol Vis Sci 48(9):3962–3967 17724173 10.1167/iovs.06-1221

[CR5] Arora A, Guduric-Fuchs J, Harwood L et al (2010) Prediction of microRNAs affecting mRNA expression during retinal development. BMC Dev Biol 10:1–15 20053268 10.1186/1471-213X-10-1 PMC2821300

[CR6] Azaiez H, Decker AR, Booth KT, Simpson AC, Shearer AE, Huygen PL, Bu F, Hildebrand MS, Ranum PT, Shibata SB, Turner A, Zhang Y, Kimberling WJ, Cornell RA, Smith RJ (2015) HOMER2, a stereociliary scaffolding protein, is essential for normal hearing in humans and mice. PLoS Genet 11(3): 10.1371/journal.pgen.1005137 25816005 10.1371/journal.pgen.1005137 PMC4376867

[CR7] Bagnoli P, Dal Monte M, Casini G (2003) Expression of neuropeptides and their receptors in the developing retina of mammals. Histol Histopathol 18(4):1219–1242 12973690 10.14670/HH-18.1219

[CR8] Bassett EA, Wallace VA (2012) Cell fate determination in the vertebrate retina. Trends Neurosci 35:565–573 22704732 10.1016/j.tins.2012.05.004

[CR9] Beer-Hammer S, Lee SC, Mauriac SA et al (2018) Gαi proteins are indispensable for hearing. Cell Physiol Biochem 47:1509–1532 29940568 10.1159/000490867 PMC11825972

[CR10] Coolen M, Katz S, Bally-Cuif L (2013) miR-9: a versatile regulator of neurogenesis. Front Cell Neurosci 7:220 24312010 10.3389/fncel.2013.00220 PMC3834235

[CR11] Crooks J, Kolb H (1992) Localization of GABA, glycine, glutamate and tyrosine hydroxylase in the human retina. J Comp Neurol 315:287–302 1346792 10.1002/cne.903150305

[CR12] Gao FB (2010) Context-dependent functions of specific microRNAs in neuronal development. Neural Dev 5:1–9 20920300 10.1186/1749-8104-5-25 PMC2958854

[CR13] Georgi SA, Reh TA (2010) Dicer is required for the transition from early to late progenitor state in the developing mouse retina. J Neurosci 30(11):4048–4061 20237275 10.1523/JNEUROSCI.4982-09.2010 PMC2853880

[CR14] Guo S, Zhang Y, Zhou T et al (2017) Role of GATA binding protein 4 (GATA4) in the regulation of tooth development via GNAI3. Sci Rep. 10.1038/S41598-017-01689-1 28484278 10.1038/s41598-017-01689-1 PMC5431507

[CR15] Hackler L, Wan J, Swaroop A et al (2010) MicroRNA profile of the developing mouse retina. Investig Ophthalmol vis Sci 51(4):1823–1831 19933188 10.1167/iovs.09-4657 PMC2868396

[CR16] Haverkamp S, Wässle H (2004) Characterization of an amacrine cell type of the mammalian retina immunoreactive for vesicular glutamate transporter 3. J Comp Neurol 468:251–263 14648683 10.1002/cne.10962

[CR17] Hoon M, Okawa H, Della Santina L, Wong ROL (2014) Functional architecture of the retina: development and disease. Prog Retin Eye Res 42:44–84 24984227 10.1016/j.preteyeres.2014.06.003 PMC4134977

[CR18] Huang S, Li Q, Alberts I, Li X (2016) PRKX, a novel cAMP-dependent protein kinase member, plays an important role in development. J Cell Biochem 117:566–573 26252946 10.1002/jcb.25304

[CR19] Johansson K, Bruun A, deVente J, Ehinger B (2000) Immunohistochemical analysis of the developing inner plexiform layer in postnatal rat retina. Investig Ophthalmol Vis Sci 41(1):305–313 10634635

[CR20] Kapsimali M, Kloosterman WP, de Bruijn E et al (2007) MicroRNAs show a wide diversity of expression profiles in the developing and mature central nervous system. Genome Biol 8(8):R173 17711588 10.1186/gb-2007-8-8-r173 PMC2375003

[CR21] Karali M, Peluso I, Gennarino VA et al (2010) miRNeye: a microRNA expression atlas of the mouse eye. BMC Genomics 11:715 21171988 10.1186/1471-2164-11-715 PMC3018480

[CR22] Kerr NM, Johnson CS, de Souza CF et al (2010) Immunolocalization of gap junction protein connexin43 (GJA1) in the human retina and optic nerve. Investig Ophthalmol Vis Sci 51:4028–4034 20375327 10.1167/iovs.09-4847

[CR23] Kolanowska M, Kubiak A, Jażdżewski K, Wójcicka A (2018) MicroRNA analysis using next-generation sequencing. Methods Mol Biol 1823:87–101. 10.1007/978-1-4939-8624-8_8 29959676 10.1007/978-1-4939-8624-8_8

[CR24] La Torre A, Georgi S, Reh TA (2013) Conserved microRNA pathway regulates developmental timing of retinal neurogenesis. Proc Natl Acad Sci USA 110(26):E2362–E2370 23754433 10.1073/pnas.1301837110 PMC3696811

[CR25] Leucht C, Stigloher C, Wizenmann A et al (2008) MicroRNA-9 directs late organizer activity of the midbrain-hindbrain boundary. Nat Neurosci 11(6):641–648 18454145 10.1038/nn.2115

[CR26] Li W, Yu Z-X, Kotin RM (2005) Profiles of PrKX expression in developmental mouse embryo and human tissues. J Histochem Cytochem 53:1003–1009 15879576 10.1369/jhc.4A6568.2005

[CR27] Linsen SEV, de Wit E, de Bruijn E, Cuppen E (2010) Small RNA expression and strain specificity in the rat. BMC Genomics 11:249 20403161 10.1186/1471-2164-11-249 PMC2864251

[CR28] Livak KJ, Schmittgen TD (2001) Analysis of relative gene expression data using real-time quantitative PCR and the 2-ΔΔCT method. Methods 25:402–408 11846609 10.1006/meth.2001.1262

[CR29] Maiorano NA, Hindges R (2012) Non-coding RNAs in retinal development. Int J Mol Sci 13(1):558–578 22312272 10.3390/ijms13010558 PMC3269706

[CR30] Masland RH (2004) Neuronal cell types. Curr Biol 14:R497–R500. 10.1016/J.CUB.2004.06.035 15242626 10.1016/j.cub.2004.06.035

[CR31] Massey SC (1990) Chapter 11 cell types using glutamate as a neurotransmitter in the vertebrate retina. Prog Retin Res 9:399–425

[CR32] Pöstyéni E, Kovács-Valasek A, Urbán P et al (2021a) Profile of miR-23 expression and possible role in regulation of glutamic acid decarboxylase during postnatal retinal development. Int J Mol Sci 22(13):7078 34209226 10.3390/ijms22137078 PMC8268301

[CR33] Pöstyéni E, Kovács-Valasek A, Urbán P et al (2021b) Analysis of mir-9 expression pattern in rat retina during postnatal development. Int J Mol Sci 22(5):1–18 10.3390/ijms22052577 PMC7961372 33806574

[CR34] Pritchard CC, Cheng HH, Tewari M (2012) MicroRNA profiling: approaches and considerations. Nat Rev Genet 13(5):358–369 22510765 10.1038/nrg3198 PMC4517822

[CR35] Qi X (2016) The role of miR-9 during neuron differentiation of mouse retinal stem cells. Artif Cells Nanomed Biotechnol 44:1883–1890 26701739 10.3109/21691401.2015.1111231

[CR36] Rapaport DH, Wong LL, Wood ED, Yasumura D, Lavail MM (2004) Timing and topography of cell genesis in the rat retina. J Comp Neurol 474:304–324 15164429 10.1002/cne.20134

[CR37] Reese BE (2011) Development of the retina and optic pathway. Vis Res 51:613–632 20647017 10.1016/j.visres.2010.07.010 PMC2974959

[CR38] Reese BE, Colello RJ (1992) Neurogenesis in the retinal ganglion cell layer of the rat. Neuroscience 46:419–429 1542415 10.1016/0306-4522(92)90062-7

[CR39] RStudio Team (2015) RStudio: integrated development for R [Computer software v0.98.1074]. RStuio Inc., Boston

[CR40] Sharma A, LeVaillant CJ, Plant GW, Harvey AR (2014) Changes in expression of Class 3 Semaphorins and their receptors during development of the rat retina and superior colliculus. BMC Dev Biol 14:34. 10.1186/s12861-014-0034-9 25062604 10.1186/s12861-014-0034-9 PMC4121511

[CR41] Sherry DM, Wang MM, Bates J, Frishman LJ (2003) Expression of vesicular glutamate transporter 1 in the mouse retina reveals temporal ordering in development of rod vs. cone and ON vs. OFF circuits. J Comp Neurol 465(4):480–98. 10.1002/cne.10838 12975811 10.1002/cne.10838

[CR42] Sundermeier TR, Palczewski K (2012) The physiological impact of microRNA gene regulation in the retina. Cell Mol Life Sci 69:2739–2750 22460583 10.1007/s00018-012-0976-7 PMC3443797

[CR43] Thoreson WB, Witkovsky P (1999) Glutamate receptors and circuits in the vertebrate retina. Prog Retin Eye Res 18:765–810 10530751 10.1016/s1350-9462(98)00031-7

[CR44] Trapnell C, Williams BA, Pertea G et al (2010) Transcript assembly and quantification by RNA-Seq reveals unannotated transcripts and isoform switching during cell differentiation. Nat Biotechnol 28:511–515 20436464 10.1038/nbt.1621 PMC3146043

[CR45] Vlachos IS, Zagganas K, Paraskevopoulou MD et al (2015) DIANA-miRPath v3.0: deciphering microRNA function with experimental support. Nucleic Acids Res 43:W460–W466. 10.1093/NAR/GKV403 25977294 10.1093/nar/gkv403 PMC4489228

[CR46] Völgyi B, Kovács-öller T, Atlasz T et al (2013) Gap junctional coupling in the vertebrate retina: variations on one theme? Prog Retin Eye Res 34:1–18 23313713 10.1016/j.preteyeres.2012.12.002

[CR47] Xiang M (2013) Intrinsic control of mammalian retinogenesis. Cell Mol Life Sci 70(14):2519–2532 23064704 10.1007/s00018-012-1183-2 PMC3566347

[CR48] Xu S, Witmer PD, Lumayag S et al (2007) MicroRNA (miRNA) transcriptome of mouse retina and identification of a sensory organ-specific miRNA cluster. J Biol Chem 282(34):25053–25066 17597072 10.1074/jbc.M700501200

[CR49] Yang XL (2004) Characterization of receptors for glutamate and GABA in retinal neurons. Prog Neurobiol 73:127–150. 10.1016/j.pneurobio.2004.04.002 15201037 10.1016/j.pneurobio.2004.04.002

[CR50] Yao M, Chen G, Zhao P et al (2012) Transcriptome analysis of microRNAs in developing cerebral cortex of rat. BMC Genomics 13:232. 10.1186/1471-2164-13-232 22691069 10.1186/1471-2164-13-232 PMC3441217

