# Supplementary material for: miRNA Profiling of Developing Rat Retina in the First Three Postnatal Weeks
Source: Cell Mol Neurobiol. 2023 Apr 21;43(6):2963–74. doi: 10.1007/s10571-023-01347-3 (PMC10333372; doi:10.1007/s10571-023-01347-3)
Supplement: Supplementary file 1 — Supplementary file1 (PDF 507 kb) [file 10571_2023_1347_MOESM1_ESM.pdf]

miRNA profiling of developing rat retina in the first three postnatal weeks

Cellular and Molecular Neurobiology

Péter Urbán1,3 urban.peter@pte.hu ORCID: 0000-0003-4043-3428
Etelka Pöstyéni[2] etelka91@gamma.ttk.pte.hu
Lilla Czuni[1] czuni.lilla@pte.hu
Róbert Herczeg1 herczeg.robert@pte.hu ORCID: 0000-0002-5903-0082
Csaba Fekete3 fekete@gamma.ttk.pte.hu
Róbert Gábriel1,2 gabriel@gamma.ttk.pte.hu ORCID: 0000-0001-6284-2420
and Andrea Kovács-Valasek2* valasek@gamma.ttk.pte.hu ORCID: 0000-0001-7021-5750
1 János Szentágothai Research Centre, University of Pécs, Pécs, Hungary
2 Experimental Zoology and Neurobiology, University of Pécs, Pécs, Hungary
3 Department of General and Environmental Microbiology, University of Pécs, Pécs, Hungary
*Contact author correspondence:
Name: Andrea Kovács-Valasek
E-mail address: valasek@gamma.ttk.pte.hu

Additional file 1.pdf: Power plot analysis to estimate sample size.

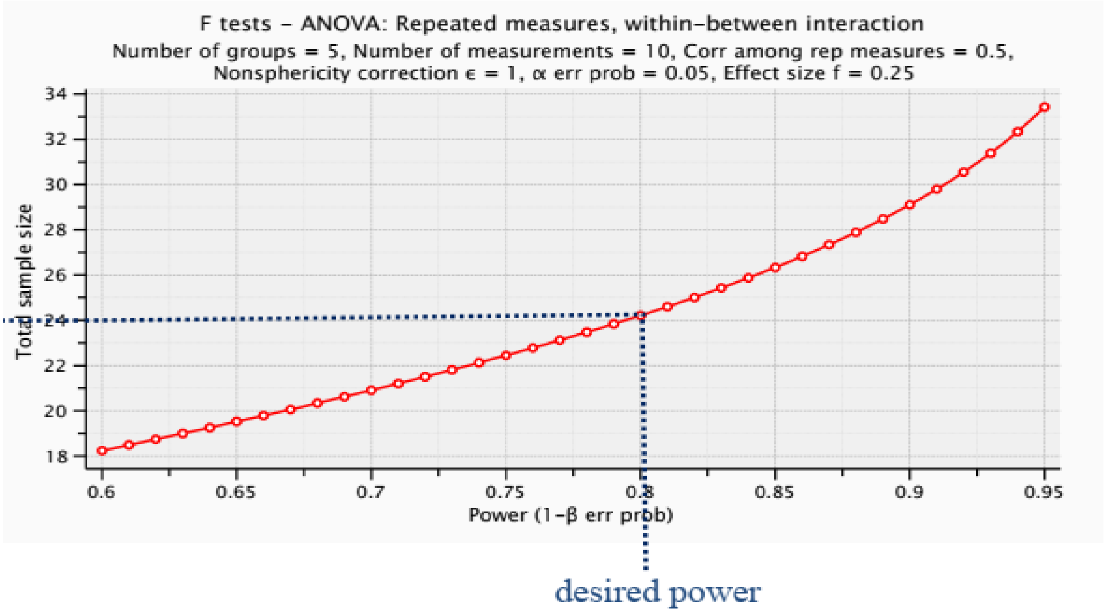

miRNA profiling of developing rat retina in the first three postnatal weeks

Cellular and Molecular Neurobiology

Péter Urbán1,3 urban.peter@pte.hu ORCID: 0000-0003-4043-3428
Etelka Pöstyéni[2] etelka91@gamma.ttk.pte.hu
Lilla Czuni[1] czuni.lilla@pte.hu
Róbert Herczeg1 herczeg.robert@pte.hu ORCID: 0000-0002-5903-0082
Csaba Fekete3 fekete@gamma.ttk.pte.hu
Róbert Gábriel1,2 gabriel@gamma.ttk.pte.hu ORCID: 0000-0001-6284-2420
and Andrea Kovács-Valasek2* valasek@gamma.ttk.pte.hu ORCID: 0000-0001-7021-5750
1 János Szentágothai Research Centre, University of Pécs, Pécs, Hungary
2 Experimental Zoology and Neurobiology, University of Pécs, Pécs, Hungary
3 Department of General and Environmental Microbiology, University of Pécs, Pécs, Hungary
*Contact author correspondence:
Name: Andrea Kovács-Valasek
E-mail address: valasek@gamma.ttk.pte.hu

Additional file 2.pdf: Quality metrics of Ion Torrent PGM Sequencings.

	quality percantage of the library	sequencing chip type	Number of bases	Number of >=Q20 bases	Number of reads	Avarage read lenghts	Histogram	Number of aligned read
P5	58.8%	316 v2	11 725 388	10 907 303	503 076	23		383 257
	46.95%	318 v2	5 357 230	3 452 602	190 689	28		123 907
P7	57.5%	316 v2	8 535 864	5 526 404	431 952	19		272 122
	57.5%	316 v2.	12 899 858	10 621 000	494 016	26		368 488
P10	65.0%	316 v2	14 372 487	12 346 040	560 847	25		429 041
	52.4%	318 v2	3 623 385	2 484 368	141 591	25		103 833
P15	73.0%	316 v2	9 948 258	8 411 439	491 292	20		338 146
	72.8%	318 v2	2 990 414	2 064 828	119 124	25		89 060
P21	57.9%	316 v2	15 616 024	14 233 199	690 771	22		527 745
	57.9%	316 v2	15 101 330	12 354 040	657 097	22		480 617
	57.9%	316 v2	13 328 951	11 540 671	592 643	22		449 416
	57.9%	318 v2	4 224 019	2 867 675	161 073	26		112 452

miRNA profiling of developing rat retina in the first three postnatal weeks
Cellular and Molecular Neurobiology
Péter Urbán1,3 urban.peter@pte.hu ORCID: 0000-0003-4043-3428
Etelka Pöstyéni[2] etelka91@gamma.ttk.pte.hu
Lilla Czuni[1] czuni.lilla@pte.hu
Róbert Herczeg1 herczeg.robert@pte.hu ORCID: 0000-0002-5903-0082
Csaba Fekete3 fekete@gamma.ttk.pte.hu
Róbert Gábriel1,2 gabriel@gamma.ttk.pte.hu ORCID: 0000-0001-6284-2420
and Andrea Kovács-Valasek2* valasek@gamma.ttk.pte.hu ORCID: 0000-0001-7021-5750
1 János Szentágothai Research Centre, University of Pécs, Pécs, Hungary
2 Experimental Zoology and Neurobiology, University of Pécs, Pécs, Hungary
3 Department of General and Environmental Microbiology, University of Pécs, Pécs, Hungary
*Contact author correspondence:
Name: Andrea Kovács-Valasek
E-mail address: valasek@gamma.ttk.pte.hu

Additional file 3.pdf: Principle component analysis among all samples.

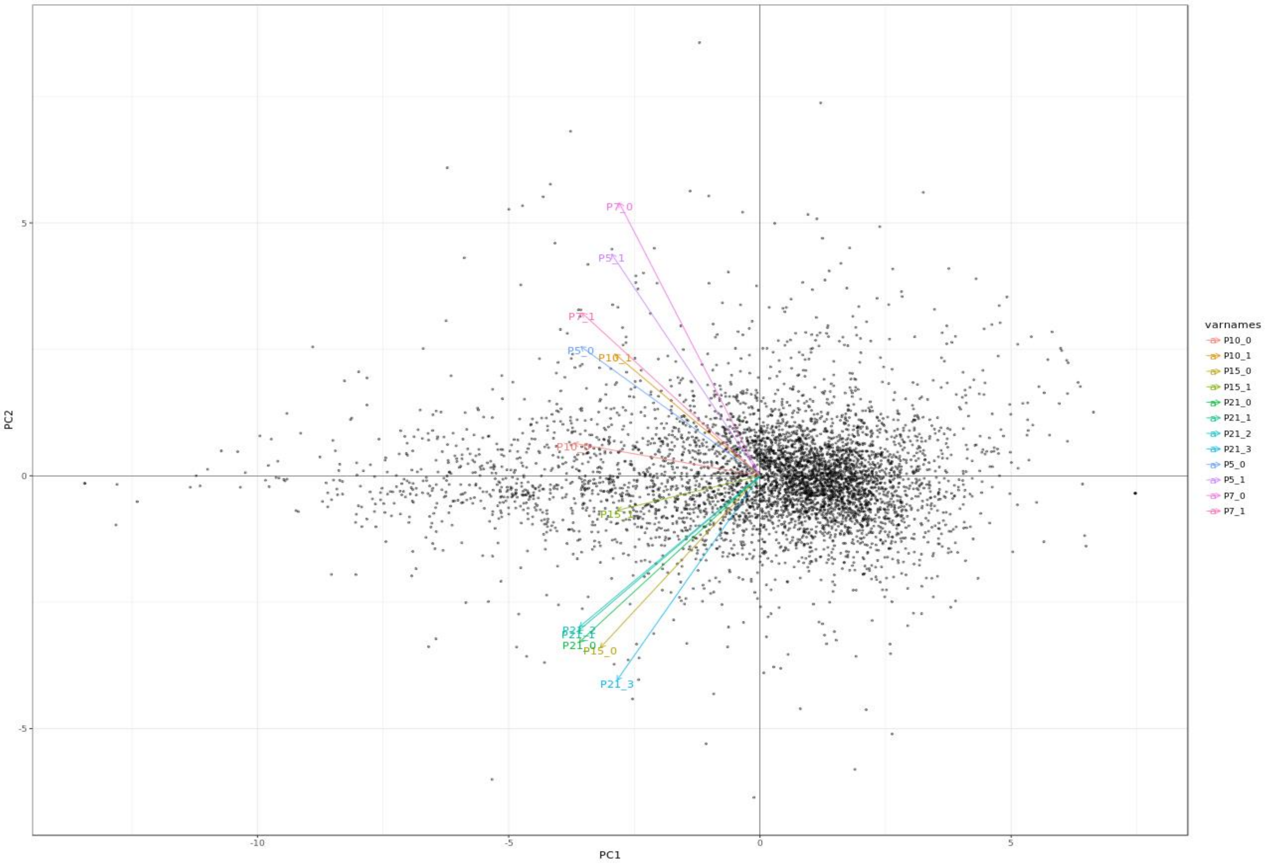

miRNA profiling of developing rat retina in the first three postnatal weeks
Cellular and Molecular Neurobiology

Péter Urbán1,3 urban.peter@pte.hu ORCID: 0000-0003-4043-3428
Etelka Pöstyéni[2] etelka91@gamma.ttk.pte.hu
Lilla Czuni[1] czuni.lilla@pte.hu
Róbert Herczeg1 herczeg.robert@pte.hu ORCID: 0000-0002-5903-0082
Csaba Fekete3 fekete@gamma.ttk.pte.hu
Róbert Gábriel1,2 gabriel@gamma.ttk.pte.hu ORCID: 0000-0001-6284-2420
and Andrea Kovács-Valasek2* valasek@gamma.ttk.pte.hu ORCID: 0000-0001-7021-5750
1 János Szentágothai Research Centre, University of Pécs, Pécs, Hungary
2 Experimental Zoology and Neurobiology, University of Pécs, Pécs, Hungary
3 Department of General and Environmental Microbiology, University of Pécs, Pécs, Hungary
*Contact author correspondence:
Name: Andrea Kovács-Valasek
E-mail address: valasek@gamma.ttk.pte.hu

Additional file 4.pdf List of differential expressed miRNAs on consecutive time points with p<0.05

gene_symbol	P5	P7	log2(fold_change)	test_stat	p_value	q_value
rno-miR-30c1	1450000	143293	-3.34	-2.4973	0.0001	0.0325
rno-miR-30c2	1870000	140671	-3.73	-2.7667	0.0001	0.0189
rno-miR-19a	9100000	3130000	-1.54	-1.1425	0.0332	0.8667
rno-miR-153	9284	3395	-1.45	-1.2093	0.0335	0.8714

gene_symbol	P7	P10	log2(fold_change)	test_stat	p_value	q_value
rno-miR-137	21999	59159	1.43	1.1245	0.0450	0.8858
rno-miR-142	7172	25031	1.80	1.4125	0.0177	0.6813
rno-miR-144	14230	65684	2.21	2.1170	0.0291	0.8472
rno-miR-148a	18058	54663	1.60	1.2574	0.0325	0.8646
rno-miR-153	3395	24878	2.87	2.2143	0.0002	0.0432
rno-miR-190b	267474	801178	1.58	1.2677	0.0289	0.8442
rno-miR-218	45801	130931	1.52	1.1617	0.0276	0.8314
rno-miR-26b	281635	787303	1.48	1.1344	0.0365	0.8858
rno-miR-30c1	143293	2620000	4.19	3.1124	0.0001	0.0189
rno-miR-30c2	140671	3270000	4.54	3.3554	0.0001	0.0189
rno-miR-32	30483	398850	3.71	2.3316	0.0012	0.5860
rno-miR-340-1	78410	276118	1.82	1.5283	0.0088	0.5210
rno-miR-340-2	115504	289815	1.33	1.1953	0.0275	0.8309
rno-miR-3565	1853547	22286	1.68	1.2844	0.0153	0.6432
rno-miR-3579	8501	43999	2.37	1.7937	0.0036	0.3312
rno-miR-3597-1	73138	256081	1.81	1.3764	0.0149	0.6390
rno-miR-3597-2	100086	342513	1.77	1.3538	0.0176	0.6799
rno-miR-3597-3	39915	137084	1.78	1.3450	0.0195	0.7194
rno-miR-384	307709	801910	1.38	1.1120	0.0385	0.8858
rno-miR-410	14845	62820	2.08	1.5597	0.0095	0.5275
rno-miR-411	80714	176295	1.13	1.0484	0.0414	0.8858
rno-miR-496	14800	40193	1.44	1.3176	0.0207	0.7398
rno-miR-758	11557	36228	1.65	2.0943	0.0441	0.8858
rno-miR-96	567553	2690000	2.24	1.7068	0.0023	0.2652

gene_symbol	P10	P15	log2(fold_change)	test_stat	p_value	q_value
rno-miR-1247	73913	23938	-1.63	-1.3919	0.0255	0.9626

gene_symbol	P15	P21	log2(fold_change)	test_stat	p_value	q_value
rno-miR-30c1	2850000	226362	-3.66	-2.9613	0.0001	0.0189
rno-miR-30c2	3990000	276814	-3.85	-3.0847	0.0001	0.0189
rno-miR-32	123819	32836	-1.91	-1.6585	0.0044	0.3768
rno-miR-153	17813	1478655	-1.58	-1.4161	0.0105	0.5567
rno-miR-340	274895	82687	-1.73	-1.4844	0.0112	0.5721
rno-miR-3579	54591	19841	-1.46	-1.2748	0.0123	0.5892
rno-miR-340	291512	100675	-1.53	-1.3515	0.0139	0.6131
rno-miR-374b	724520	237549	-1.61	-1.3588	0.0170	0.6742
rno-miR-184	89169	242687	1.44	1.1299	0.0487	0.8858

miRNA profiling of developing rat retina in the first three postnatal weeks

Cellular and Molecular Neurobiology

Péter Urbán1,3 urban.peter@pte.hu ORCID: 0000-0003-4043-3428
Etelka Pöstyéni[2] etelka91@gamma.ttk.pte.hu
Lilla Czuni[1] czuni.lilla@pte.hu
Róbert Herczeg1 herczeg.robert@pte.hu ORCID: 0000-0002-5903-0082
Csaba Fekete3 fekete@gamma.ttk.pte.hu
Róbert Gábriel1,2 gabriel@gamma.ttk.pte.hu ORCID: 0000-0001-6284-2420
and Andrea Kovács-Valasek2* valasek@gamma.ttk.pte.hu ORCID: 0000-0001-7021-5750
1 János Szentágothai Research Centre, University of Pécs, Pécs, Hungary
2 Experimental Zoology and Neurobiology, University of Pécs, Pécs, Hungary
3 Department of General and Environmental Microbiology, University of Pécs, Pécs, Hungary
*Contact author correspondence:
Name: Andrea Kovács-Valasek
E-mail address: valasek@gamma.ttk.pte.hu

Additional file 5.pdf: DIANA miRPath analysis and KEGG pathway enrichment constructed by differentially expressed miRNAs on consecutive time-points with log2 fold change ≤ or ≥ ± 2 on the DIANA-TargetScan algorithm. The most relevant categories for retinal metabolism were labelled by yellow.

		KEGG category	p-value	genes	miRNAs
		**P5_P7**			
Cellular community	1	Gap junction (rno04540)	0.00	4	4
Lipid	2	Biosynthesis of unsaturated fatty acids (rno01040)	0.0189348	1	1
Nervous system	3	Glutamatergic synapse (rno04724)	0.0189348	4	4
Cancer	4	Pathways in cancer (rno05200)	0.0189348	12	7
Substance dependence	5	Cocaine addiction (rno05030)	0.0262045	2	3
Amino acid	6	Valine, leucine and isoleucine degradation (rno00280)	0.0268587	2	1
		**P7_P10**			
Lipid	1	Arachidonic acid metabolism (rno00590)	0.04	2	2
Glycan	2	N-Glycan biosynthesis (rno00510)	0.0476933	2	2
		**P10_P15**			
Excretory system	1	Endocrine and other factor-regulated calcium reabsorption (rno04961)	1.01E-06	1	1
Glycan	2	Other glycan degradation (rno00511)	1.44E-06	1	1
Glycan	3	Glycosphingolipid biosynthesis - lacto and neolacto series (rno00601)	1.44E-06	1	1
Glycan	4	Mucin type O-Glycan biosynthesis (rno00512)	4.88E-06	2	1
Lipid	5	Glycerophospholipid metabolism (rno00564)	6.40E-06	4	3
Amino acid	6	Lysine degradation (rno00310)	0.031793	2	2
Amino acid	7	Tyrosine metabolism (rno00350)	0.0440768	1	1
		**P15_P21**			
Glycan	1	Glycosphingolipid biosynthesis - lacto and neolacto series (rno00601)	0.00	1	1
Glycan	2	Mucin type O-Glycan biosynthesis (rno00512)	3.92E-09	2	1
Glycan	3	Other glycan degradation (rno00511)	5.81E-09	1	1
Lipid	4	Glycerophospholipid metabolism (rno00564)	0.0014074	2	1
Metabolic	5	Metabolic pathways (rno01100)	0.002011	17	3
Cofactors vitamins	6	Nicotinate and nicotinamide metabolism (rno00760)	0.0063398	1	1
Glycan	7	Glycosylphosphatidylinositol(GPI)-anchor biosynthesis (rno00563)	0.0183713	2	2
Digestive	8	Vitamin digestion and absorption (rno04977)	0.0220289	2	1
